# Multimodality Imaging of the Anatomy of Tricuspid Valve

**DOI:** 10.3390/jcdd8090107

**Published:** 2021-09-03

**Authors:** Susanne Anna Schlossbauer, Francesco Fulvio Faletra, Vera Lucia Paiocchi, Laura Anna Leo, Giorgio Franciosi, Michela Bonanni, Gianmarco Angelini, Anna Giulia Pavon, Enrico Ferrari, Siew Yen Ho, Rebecca T. Hahn

**Affiliations:** 1 Cardiocentro Ticino Institute, Ente Ospedaliero Cantonale, 6900 Lugano, Switzerland; susanne.schlossbauer@eoc.ch (S.A.S.); vera.paiocchi@eoc.ch (V.L.P.); lauraanna.leo@eoc.ch (L.A.L.); franciosigiorgio1351@hotmail.com (G.F.); Michela.bonanni@eoc.ch (M.B.); gianmarco.angelini@eoc.ch (G.A.); Annagiulia.pavon@eoc.ch (A.G.P.); enrico.ferrari@eoc.ch (E.F.); 2 Cardiac Morphology Unit, Royal Brompton Hospital, London SW36NP, UK; Yen.ho@imperial.ac.uk; 3 Cardiovascular Research Foundation, New York Presbyterian Hospital, Columbia University Medical Center, New York, NY 10032, USA; rth2@cumc.columbia.edu

**Keywords:** tricuspid valve, tricuspid valve insufficiency, multimodality imaging

## Abstract

Even though the tricuspid valve is no longer “forgotten”, it still remains poorly understood. In this review, we focus on some controversial and still unclear aspects of tricuspid anatomy as illustrated by noninvasive imaging techniques. In particular, we discuss the anatomical architecture of the so-called tricuspid annulus with its two components (i.e., the mural and the septal annulus), emphasizing the absence of any fibrous “ring” around the right atrioventricular junction. Then we discussed the extreme variability in number and size of leaflets (from two to six), highlighting the peculiarities of the septal leaflet as part of the septal atrioventricular junction (crux cordis). Finally, we describe the similarities and differences between the tricuspid and mitral valve, suggesting a novel terminology for tricuspid leaflets.

## 1. Introduction

Today, the major impact on strategic management of tricuspid valve (TV) regurgitation (TR) is still dictated still by the work of Nina Braunwald et al., which reviewed the clinical outcome of 100 patients who underwent mitral replacement in 1967 [1].

Among them, 28 patients had clinical and hemodynamic evidence of significant TR at the time of mitral replacement for either mitral stenosis or regurgitation, but an associated surgical procedure to correct TR was performed only in three cases. The authors followed up the 24/28 survivors of the operation for a time interval of 1 to 4 years (average 30 months), and all experienced clinical improvement with a reduction in mean right atrial pressure (from 11 mm Hg to 5 mmHg) and mean systolic pulmonary pressure (from 75 mmHg to 39 mmHg). They concluded that secondary TR “will improve or disappear after mitral replacement and that TV replacement is seldom necessary”. Needless to say, nowadays this paper would undergo severe criticism: the patient population was likely rheumatic (though not explicitly stated), and only 28 patients (and 24 operative survivors) were included in the study with a short follow up time (average 30 months). Moreover, the follow up consisted only of clinical examination and the assessment of right atrial and mean systolic pulmonary pressure, without an objective tool to evaluate the residual TR and right ventricular (RV) size and function. In the discussion, the authors state that “symptomatic improvement in the later postoperative period seemed to be achieved more rapidly by patients without than those with TR”; however, they discounted this as the “impression” of the treating physicians.

In those days, the study was highly regarded for several reasons; first and foremost because it gave a reason for the surgeons to avoid implanting a second prosthesis that would be deemed useless. From then onwards, the TV pathology was considered a “benign” disease and somehow “forgotten” by the cardiology community.

A renewed interest on TV was elicited by a study published by Nath et al. in 2004 [2]. The authors followed up 5223 patients for four years with variable degrees of TR. They found that mortality increased with increasing degrees of TR severity. One-year survival was 90.3% in patients with mild TR, 78.9% in patients with moderate TR, and 63.9% in cases of severe TR, regardless of association with left ventricular disfunction or pulmonary hypertension. This study had the same impact albeit in the opposite direction from that of Braunwald et al. and was followed by several others which confirmed Nath’s results [3,4,5,6]. Eventually, cardiologists realized that functional TR was not an innocuous “bystander” of left sided valvular disease which disappears (or at least significantly decreases) after left valve repair/replacement. Instead, it is an insidious, unpredictable and often irreversible disease, which, if left untreated, progressively may lead to RV failure, a poor quality of life and eventually to death.

### 1.1. Prevalence of TR

The prevalence of TR has been poorly known for a long time. The main reason is that many proven significant TR are clinically undetectable. Thus, 2D TTE color Doppler remains the main tool for detecting TR and define its prevalence. Cahill et al. [7] analyzed data from the OxValve study consisting of a large (>4000) representative sample of subjects of age ≥65 who were screened specifically for valve disease. Overall, 69/4009 (1.7%, 95% CI 1.4% to 2.2%) subjects screened had a new diagnosis of moderate to severe TR, which represents 44,5% of all cases of TR (69/155). The prevalence was strongly age related, with a raw prevalence of 1.1% (95% CI 0.8% to 1.6%) in subjects aged 65–74 years, and 6.6% (95% CI 5.5% to 7.8%) in those aged ≥75 years. Topilsky et al. [8] analyzed a subset of 21,020 echocardiograms performed at Mayo Clinic over a 10-year period. This geographically-well-defined study population (Olmsted County, Minnesota) allowed detection of almost all cases of TR diagnosed in the community. The study indicates a TR overall prevalence of 0.55% [0.50–0.60], approximately one-fourth of all left-sided valve disease, and similar to the prevalence of aortic stenosis. Of note, the observed survival in patients with moderate-to-severe TR was 28.5 ± 1.3% at 5 years, 14.1 ± 1.1% at 10 years, 10.2 ± 1.1% at 15 years, significantly lower than in patients with trivial TR (*p* < 0.0001). Thus, in agreement with the study published by Nath et al., greater or equal to moderate isolated TR has an elevated risk of mortality. Indeed, adjusted for age, sex, AF, ejection fraction, presence of restrictive physiology, mitral regurgitation grade, and systolic pulmonary pressure, in the Topilsky study, the risk of mortality was of 1.17 (95% CI: 1.02 to 1.22; *p* = 0.01). Finally, in both above-mentioned studies, the most frequent cause of TR was functional. The prevalence of functional TR over the organic (or primary) TR has been recently confirmed by Vieitez et al. [9]. In their prospective study of patients with significant TR, the authors found that primary TR was present in 7.4% of patients, whereas functional TR was present in 92.6%.

### 1.2. Therapeutic Options

Patients with severe TR may be asymptomatic or paucisymptomatic for a long time before facing right ventricular dysfunction. When symptomatic, medical treatment (primarily based on diuretics) may alleviate symptoms, and currently, most patients with severe TR are treated conservatively. The preference of medical therapy is due to the high morbidity and mortality risk associated with TV surgery, evaluated at 10% and 40% respectively in all major trials. [10,11,12]. Moreover, surgery is not associated with an improved long-term survival compared to medical management alone [13]. Finally, the optimal timing of TV repair, in patients undergoing left valve surgery, remains controversial, and, according to current guidelines, surgical intervention is recommended (Class I B-NR) in patients with severe TR (stages C and D) undergoing left-sided valve surgery [14,15]. At the same time, the awareness of the dismal outlook of the disease has fostered a more aggressive attitude towards intervention, with surgeons supporting tricuspid annuloplasty concomitant to left-valve surgery, in case of dilated tricuspid annulus >40 mm, regardless of TR severity [16]. Indeed, if left untreated, even mild or moderate TR may progress over time in approximately 25% of patients, affecting long-term survival [17,18]. Whether early surgical treatment reduces TR progression and reduces mortality is being tested by the Evaluating the Benefit of Concurrent Tricuspid Valve Repair During Mitral Surgery Trial (ClinicalTrials.gov Identifier: NCT02675244) which enrolled 401 patients and is awaiting completion of their two-year endpoint.

The substantial failure of an effective surgical treatment for isolated TR has accelerated the development of devices and techniques for percutaneous tricuspid interventions [18,19,20]. Preliminary results show that in selected high-risk patients with severe TR, percutaneous treatment is associated with greater survival and reduced rehospitalization for heart failure compared to medical therapy alone [21].

For a successful outcome of these novel percutaneous techniques, an in-depth understanding of the peculiar tricuspid anatomy is essential. Over the last 10 years, a significant number of papers have been published describing the TV anatomy. [22,23,24,25,26,27,28,29].

Previous studies [30,31] from our group systematically described the anatomic components of the mitral valve apparatus and aortic root through noninvasive imaging techniques [i.e., transthoracic (TTE) and transesophageal (TEE) echocardiography, cardiac tomography (CT) and cardiac magnetic resonance (CMR)]. Because of the large number of anatomical studies available, in this review we focus on some controversial or still unclear aspects of tricuspid anatomy.

In this respect, we pose the following questions:

(a) Which is the best noninvasive imaging technique to visualize TV?
(b) Does the tricuspid annulus exist?
(c) How many leaflets has the valve?
(d) Which are the similarities and differences between tricuspid and mitral valve?

## 2. Which Is the Best Noninvasive Imaging Technique to Visualize TV?

Describing and illustrating cardiac anatomy through noninvasive imaging techniques is a novel method with considerable advantages compared to cadaveric anatomic specimens. Indeed, these techniques show a kind of “live-anatomy” which couples morphology and function admirably. CT, CMR and 2D-3D TTE/TEE are usually requested to illustrate and clarify pathological states. Thus, imaging normal anatomic structures is a sort of “beneficial collateral effect” of these techniques. The same data set that show pathological features is able to consistently show anatomical details of the other normal cardiac components. Moreover, they play an essential role before, during and after both surgical and percutaneous intervention on TV. Not all these techniques are equally able for describing the anatomy of TV. In this section, we briefly illustrate their strengths and weaknesses.

### 2.1. CT Scan

Routinely, CT scan is required for evaluating coronary artery disease and pathologies of the aorta (aneurysm, dissection) [32], for selection and preprocedural planning before transcutaneous aortic valve implantation (i.e., annular size, degree and distribution of calcification and availability of the access route) [33], for a detailed anatomical assessment of left atrial appendage in patients undergoing left atrial appendage device closure [34], and, more recently, for preprocedural examination before Cardioband procedure and transcatheter mitral valve implantation [35,36]. Acquisition of left heart images with CT follows a precise temporal protocol. Consequently, in routine CT examinations, the right atrium and right ventricle are usually poorly contrasted, and the TV is almost invisible. Indeed, a clear visualization of the TV requires an opacification of the space around the valve which can be obtained only with a dedicated acquisition protocol (when the first pass contrast is in right side cavities) [37]. The main advantage of CT, in illustrating TV anatomy, is the ability to visualize the well opacified left structures around the tricuspid annulus (TA) such as the right coronary artery. However, when mistakenly or for research purposes the right structures are well opacified, the images of the TV are of exquisite quality. The x-ray exposure remains the main disadvantage, even though the newest generation CT scanners have allowed for substantially shorter scan times, less iodinated contrast, and lower radiation dose [38].

### 2.2. CMR

The ability to dynamically visualize the heart without any limitations due to body habitus and the absence of ionizing radiation makes CMR an ideal imaging technique [39]. However, due to spatial resolution, studying TV leaflets with CMR may be challenging; blurring and ghost artifacts not rarely may deteriorate image quality. Nevertheless, the ability of steady-state free-precession (SSFP) sequences to obtain strong signal from fat and blood and weak signal from myocardium enables clarification of specific anatomic details of the tricuspid annulus and the pyramidal space [29]. Needless to say, CMR is the gold standard for evaluating right ventricular size and function and may be useful in grading TR in challenging cases.

### 2.3. Echocardiography

**Figure 1:**
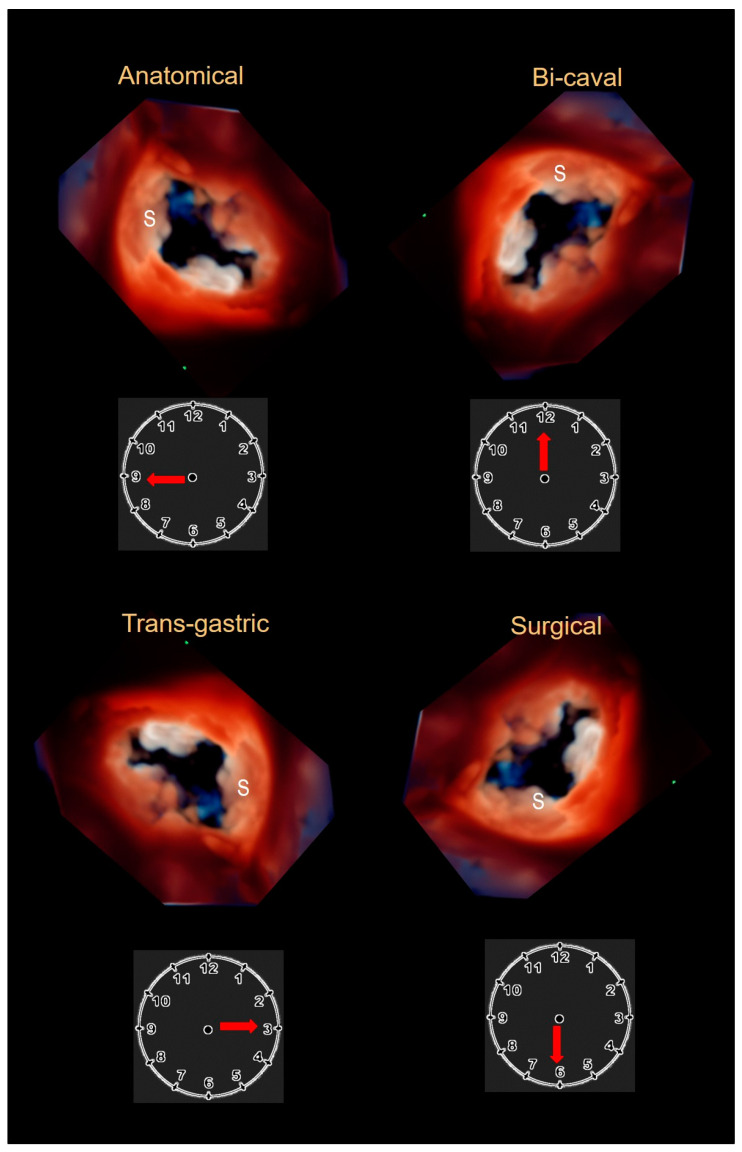
The four classic en face views of tricuspid valve. S = septal leaflet.

Echocardiography remains the first-line technique in the evaluation of TV, and it is a key modality for distinguishing primary (organic) from secondary (functional) TR. 2D TTE and 2D TEE cross sections have been described and standardized [23,39,40,41,42]. Moreover, this is the only imaging technique available for intraprocedural guiding of the novel transcatheter tricuspid procedures [42]. However, like CT and CMR, 2D TTE and TEE are tomographic techniques. Thus, usually only two of the three leaflets may be visualized in a single image (with the exception of short axis subxiphoid or transgastric cross sections views). On the contrary, 3D TTE and 3D TEE are able to visualize the entire valve in an en face perspective. The ability of a 3D data set to be rotated and cropped allows viewing of tricuspid leaflets most commonly from atrial or ventricular perspectives and also from any oblique perspective. 3D TTE and TEE provide the precise assessment of size and shape of the tricuspid annulus, number of leaflets and play an increasing role during catheter-based procedures [29,43]. Interestingly, how best to display the valve in 3D remains controversial. Having as anatomical reference the septal leaflet (which is easily recognizable), there are four classic “en face” atrial perspectives: the anatomical perspective where the septal leaflet is located at nine o’clock, the bi-caval perspective where the septal leaflet is located at twelve o’clock, the transgastric view where the septal leaflet is located at three o’clock and finally the surgical view where the septal leaflet is located at six o’clock (Figure 1).

It must be said that when using 3D TTE, the leaflets may appear thicker than they actually are, due to the poor lateral and azimuthal resolution, whereas using 3D TEE, the leaflets may suffer drop-out artifacts due to their thinness and oblique orientation with respect to the direction of ultrasound beams [42].

## 3. Does the Tricuspid Annulus Exist?

**Figure 2:**
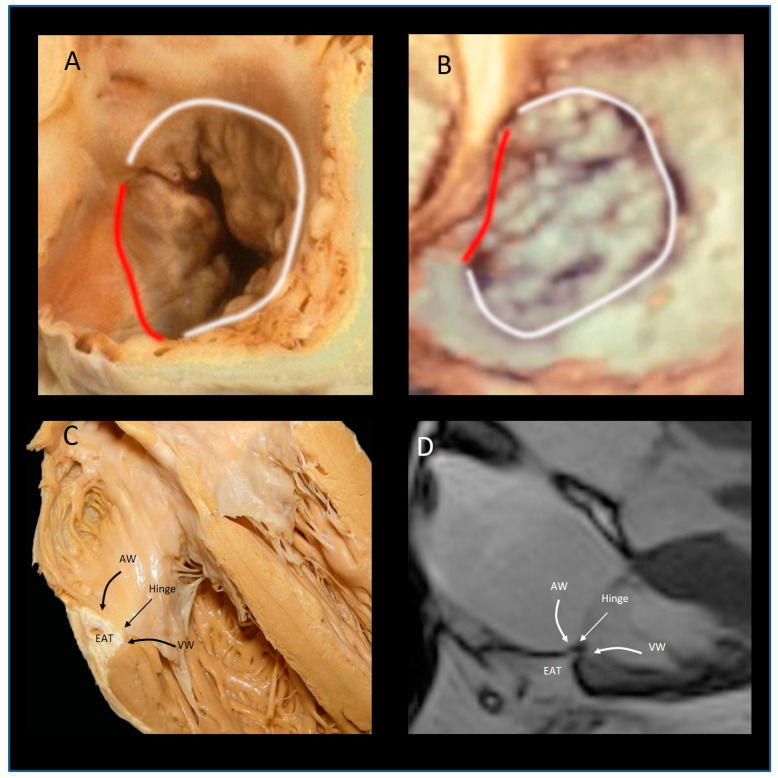
(**A**,**B**) the “oval” shape of tricuspid annulus. (**A**) anatomic specimen, (**B**) corresponding 3D TEE image. The white line marks the mural annulus, the red line the septal annulus, (**C**) cross-sectional cut of the anatomical specimen showing the four components of the mural annulus. (**D**) The corresponding CMR image. EAT = epicardial adipose tissue; VW ventricular wall; AW = atrial wall.

There is much interest in the anatomical architecture of the so-called “tricuspid annulus” (TA). Indeed, the most common cause of TR is not the anatomical involvement of tricuspid leaflets, but rather the enlargement of TA (i.e., functional TR). For this reason, the TA is the main therapeutic target for surgical treatment of functional TR and, more recently, for percutaneous annuloplasty. As seen from right atrium in empty cadaveric specimen the TA appears oval in configuration (Figure 2A,B) and significantly larger than the mitral annulus. Clinical imaging has revealed that the TA has a saddle-shaped configuration that mirrors the well-known nonplanarity of mitral annulus [44,45]. This shape has been demonstrated to reduce mitral leaflet stress [46]. However, the saddle-shaped configuration of TA is less accentuated and less defined than that of mitral annulus with peaks near the anterior-medial and posterior-medial regions and the valleys near the anterior-lateral and the posterior-medial regions. Moreover, the right ventricular pressure is one fifth that of left ventricular pressure, and consequently, the stretch effect on tricuspid leaflets is less significant than that on mitral leaflets.

The TA can be divided into two distinct segments [29], a C-shaped segment, which corresponds to the free wall of the right ventricle hosting the insertion of anterior and posterior leaflets, and a shorter straight portion, which corresponds to the insertion of the septal leaflet. These segments, named “mural” and “septal” respectively, have a close resemblance with the posterior and anterior segments of the mitral annulus and will be described separately. For consistency with the previous literature, we maintain the term of TA, describing this portion as mural and septal “annulus”.

### The “Mural” Annulus

Messer et al. studied 12 human hearts from patients who died following cardiac surgery and underwent autopsy. They found no significant amount of fibrous tissue along the C-shaped mural segment [47]. In other words, the mural portion is comprised of the convergence of the inferior (apical) margin of the atrial wall, superior (atrial) margin of ventricular wall, the insertion of the leaflets and epicardial adipose tissue penetrating from the atrio-ventricular groove; this assembly is covered internally by a layer of endothelial cells. The noninvasive imaging techniques (especially CMR) beautifully reveal this peculiar architecture (Figure 2C,D).

**Figure 3:**
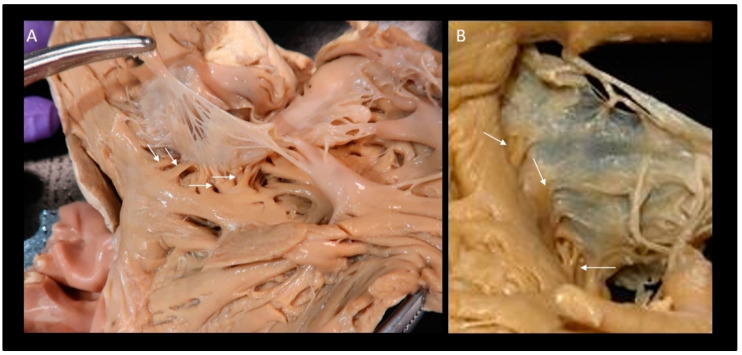
(**A**,**B**) Two anatomic specimens showing the thin muscular bars (arrows) extending from right ventricular wall to the ventricular aspect of the hinge line of leaflets.

Several thin muscular bars extend from the right ventricular wall to the ventricular aspect of the hinge line of leaflets in a crisscross fashion. These muscular bars that look like the third order chordae of mitral valve are believed to reinforce this segment of the annulus [47]. However, arising from the ventricular myocardium, they may play a role exerting a traction on the annulus in the case of an enlarged right ventricle (Figure 3). Interestingly, the insertion of the tricuspid leaflets on the mural annulus varies from patient to patient and from different areas of the mural annulus, being inserted on the apical margin of the atrial wall, on the atrial margin of ventricular wall or on both [47]. Finally, the fibrosa of the TV tapers out in the adipose tissue of the atrioventricular sulcus. This configuration contributes to the electrical insulation between right atrium and right ventricle. This atrio-ventricular plane of insulation is crossed only by the specialized muscular fibers of the atrio- ventricular conduction system.

## 4. The “Septal” Annulus

The septal annulus is made up only by the hinge line of the septal leaflet which is inserted on the ventricular myocardium, lining the inferior margin of the so-called atrio-ventricular septum (see below). Histologically, fibers of connective tissue may reinforce the hinge line especially in the region of the membranous septum, making this the most recognisable part of the annulus. As for the mural annulus, muscular thin bars arising from the septal ventricular wall insert at the hinge line of the leaflet. The septal leaflet is inserted apically relative to the insertion of the anterior mitral leaflet. In a normal heart, the distance between the two hinge lines does not exceed 8–9 mm [48]. This configuration can be appreciated in four chamber view forming the so-called “septal atrioventricular junction”.

In conclusion, though we continue to call the right atrioventricular junction tricuspid annulus, we must emphasize that, from an imager’s point of view but also from an anatomical perspective, an annulus, as assumed to be a complete fibrous ring surrounding the right atrio-ventricular orifice, does not exist. The TA corresponds to the circumference where leaflets are inserted. Accordingly, regardless of the imaging modality used, all the measurements of the annulus are taken using this hinge line.

It is interesting to note that surgeons and interventionalists propose a different segmentation of the TA. Surgeons divide the TA in four portions: the aortic, anterior, posterior and septal segments [29]. The aortic segment is from a surgical point of view, the most relevant. It consists of the hinge line of the anterior part of the septal leaflet, the hinge line of anterior leaflet and the antero-septal commissure. Its relevance is due to the fact that only a few millimeters of tissue separate this segment from the aortic root. Thus, an imprecise suture during surgical replacement with a valve prosthesis or repair with a prosthetic ring may injure the aortic leaflet or the right aortic coronary sinus, or even impinge upon the atrioventricular conduction bundle. Interventionalists, on the other hand, [49] suggested a different segmentation dividing the annulus in five areas: the antero-septal commissural area, the antero-leaflet area, the antero-posterior commissural area, the posterior segment area, and the postero-septal commissural area. This subdivision has been proposed as a practical guide during percutaneous TV repair procedures.

**Figure 4:**
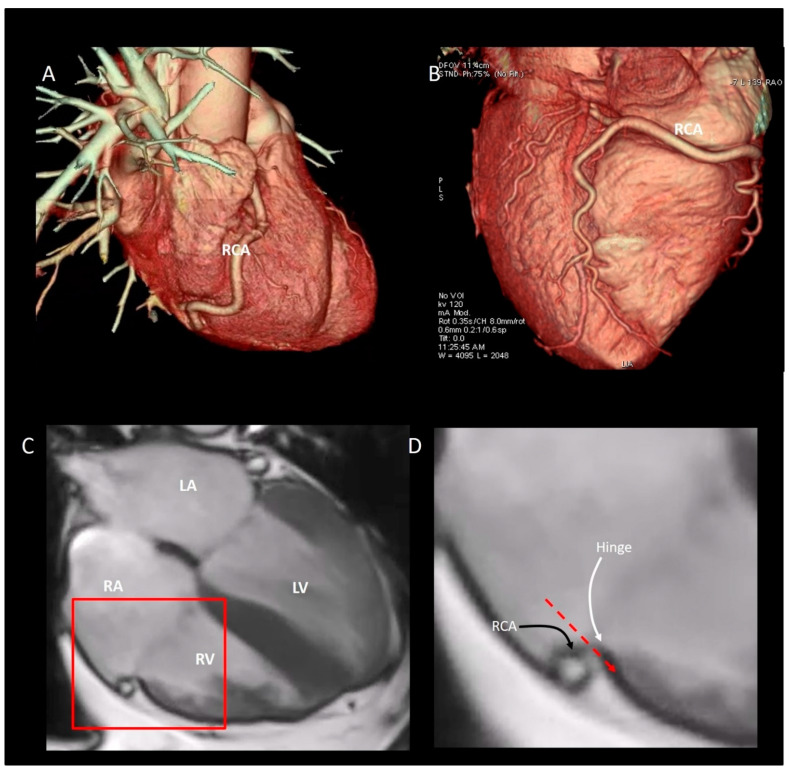
CT scan volume rendering showing the undulating course of right coronary artery (RCA). The RCA is visualized from lateral (**A**) and posterior (**B**) perspectives. (**C**) CMR four chamber view. (**D**) Magnified image of the structures inside the red square of (**C**), showing the close proximity between the hinge line of leaflet (white curved arrow) and the RCA (black curved arrow). The red dotted line marks the direction of the anchors used for the percutaneous implant of a prosthetic ring. RA = right atrium; LA = left atrium; RV = right ventricle; LV = left ventricle.

The anatomical relationship between the right coronary artery (RCA) and the mural annulus is significant. Encircling the mural annulus, the distance between RCA and the leaflets’ hinge line varies with a minimum of 5–10 mm at the posterior/septal leaflet commissure to the maximum of 20 mm at the anterior/septal leaflet commissure [50]. Importantly, the distance of the RCA to the annulus is dynamic and differs depending on the cardiac cycle, as well as the pathology of the annular anatomy. Furthermore, the course of the RCA within the atrio-ventricular groove may have an undulating course (Figure 4A,B). This dynamism may not be an issue for the surgeon operating on a flaccid heart and direct injury by acute entrapment of the RCA by surgical sutures is rare but potentially life threatening. The temporal variability of the annulus-to-RCA distance is more of an issue for beating-heart operations or interventions. The nonfibrous structure of the mural annulus may create other difficulties in some percutaneous catheter-based procedures. In order to avoid injuring the base of the leaflets, the anchoring systems used in percutaneous implant of prosthetic annulus must be inserted in between the hinge line and RCA. The RCA can be visualized with selective coronary angiography with the base of leaflets by 2D TEE. However, in absence of a robust fibrous annulus, the anchors inevitably must cross the assembly of atrial wall and epicardial adipose tissue to be inserted on the crest of ventricular myocardium (Figure 4C,D).

## 5. Physiological and Physiopathologic Consequences of the Annular Structure

**Figure 5:**
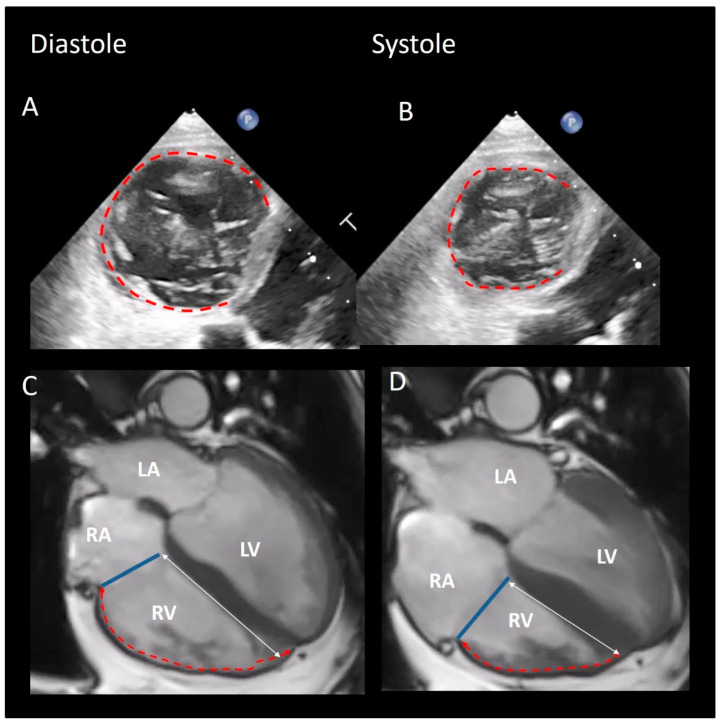
(**A**,**B**) 2D TEE showing the vigorous systolic sphincteric contraction of the mural annulus (red dotted line). (**C**,**D**) CMR in four chamber view showing the systolic translation of the annulus (blue line) towards the apex. The septal annulus has a minor systolic excursion (double head white arrow) while the mural annulus is shortening vigorously (red dotted line).

The TA has two main systolic motions: the sphincteric contraction and the excursion towards the right ventricular apex. The sphincteric contraction is prevalently due to the muscular structure of the mural annulus which follows the contraction of ventricular and atrial cavities. In the setting of a relatively fixed septal annulus, both the sphincteric action and longitudinal motion are therefore asymmetric, involving predominantly the anterior, lateral and inferior segments of the TA (Figure 4). The normal TA area in mid systole is 7.6 ± 1.7 cm^2^/m^2^, and the TA area shortens by 35% ± 10% during the cardiac cycle [44]. The excursion of TA towards the apex reflects the longitudinal contraction of the oblique-oriented subendocardial muscular layers of RV. Either CMR (Figure 4) or 2D TTE can show the longitudinal motion of the annulus. Interestingly, the annular motion is hinge-like; the septal annulus is more stable and, during the systole, has a minor systolic excursion. On the contrary, the rest of the annulus (mural annulus) shortens vigorously (Figure 5).

The annular dilatation is also asymmetric involving almost exclusively the “soft” mural annulus. Currently, two mechanisms can be described for explaining dilation of TA (and consequent TR): the “atriogenic” (previously labelled as idiopathic annular dilatation) and the “ventriculogenic” mechanisms.

In some patients, longstanding atrial fibrillation has been associated with annular dilation and TR despite the fact that the right ventricle has a normal size, shape and function [51]. The increase in the right atrial volume due to the atrial arrhythmias appears to be the main mechanism for annular dilation. Indeed, the musculature of the right atrium is thinner than that of the left atrium, and in the setting of atrial fibrillation, this cavity usually enlarges more than the corresponding left cavity, leading to a more accentuated TA dilation [51]. However, not all patients with atrial fibrillation and increased right atrial volume develop significant TR. As mentioned above, the mural annulus is formed by the juxtaposition of the atrial and ventricular myocardium, hinge line of tricuspid leaflets, and adipose tissue. The insertion of leaflets on atrial and ventricular myocardium is undulating, varying significantly between individual heart, within the same heart and between the mural and septal segments [47]. It can be speculated that in those patients in whom the hinge line of leaflets is predominantly inserted on the atrial myocardium, the right atrial enlargement may cause a kind of “disproportionate” annular dilation, leading to significant TR.

The “ventriculogenic” mechanism of TR is more complex and mirrors that of the symmetric patterns of functional mitral regurgitation. The increase in right ventricular volume disrupts the normal spatial relationships between leaflets, chordae, and papillary muscles leading to leaflet tethering, dilatation and flattening of the TA in a deleterious vicious circle. In particular, both anterior and inferior papillary muscles (PMs) move laterally and apically. The first tethers the anterior leaflet and the anterior part of the septal leaflet, whereas the second tethers the inferior leaflet and the inferior-lateral commissure. The above-mentioned thin muscular strings arising from the base of right ventricle and inserting on the annulus may contribute to the annular dilation when the right ventricle is enlarged.

In ventriculogenic TR, dilatation of the right ventricle resulting in displacement of the papillary muscles results in leaflet tethering or tenting, and apical leaflet coaptation. In the atriogenic TR, lack of leaflet coaptation is due to the annular dilation without leaflet tethering [51].

## 6. How Many Leaflets Has the Valve?

In the classic anatomy books, the TV has been traditionally described as having very distinct three leaflets with three main indentations. Using an attitudinal-appropriate nomenclature (i.e., relative to their anatomical position in the body), the leaflets can be named septal, anterior-superior and inferior. From here on, we use this nomenclature. In the classic three-leaflet configuration, the indentations (called also commissures) are located in anterior-septal position (dividing the septal from the anterior leaflet), in anterior-inferior position (dividing the anterior from the inferior leaflet), and inferior-septal position (dividing the inferior from the septal leaflet). Interestingly, mirroring the mitral commissures, the indentations do not reach the hinge line, leaving a strip of valvular tissue between the leaflets. Thus, from an anatomical point of view, the leaflets are formed by a continuous veil divided by three (or more) indentations. Each leaflet can be roughly divided in three zones: the basal zone near the hinge line, a thin and translucent “clear” zone (which corresponds to the body of the leaflet) and a rough zone towards the free edge, where chordae tendineae are inserted on its ventricular side. This rough zone corresponds to the atrial side as the coaptation area [52].

Despite the name “tricuspid”, the valve shows a large variability in terms of size and number of leaflets, and publications present divergent and often confounding results. In 1971, Silver et al. studied 50 normal heart specimens, recognizing that in 70% of patients, the inferior leaflet was scalloped, containing accessory leaflets [53]. Skwarek et al. [54], studying 107 formalin-fixed adult human hearts, found a bicuspid valve in 3%, tricuspid in 10%, quadricuspid in 41%, pentacuspid in 34%, and hexacuspid in 8% of cases. Kocak et al. [53] collecting 400 autopsy cases, found 2 leaflets in 36 hearts (18%), 3 leaflets in 130 hearts (65%), and 4 leaflets in 34 hearts (17%). Lama et al. [55] in 36 adult formalin-fixed human hearts found that five-leaflet configuration was the more common form, while the classic three-leaflets with no accessory leaflets, was only present in a small percentage of the cases. Holda et al. [56] found a “3-leaflet” configuration in 57.5% and “4-leaflet” configuration in the remaining hearts. In the 4-leaflet valves, extra leaflets were commonly observed in the inferior region of the annulus. Sakon et al. [57] found that approximately half of the tricuspid valves had multiple scallops localized in the inferior/posterior leaflet. Conversely, Kujur et al. [58] found three leaflets in all 42 formalin-fixed adult human hearts dissected. Recently, Hahn et al. [59] introduced a novel nomenclature system for the description of the number of tricuspid leaflets and for determining the relative incidence of each morphological type. Using a comprehensive TEE with three levels of imaging acquisition (mid-esophageal, distal esophageal and transgastric view), the authors identified four morphological types: type I, 3 leaflets; type II, 2 leaflets; type IIIA, 4 leaflets with 2 anterior; type IIIB, 4 leaflets with 2 posteriors; type IIIC, 4 leaflets with 2 septal; and type IV > 4 leaflets. The incidence of type I morphology was 312 of 579 (53.9%), type II was 26 of 579 (4.5%), type IIIA was 15 of 579 (2.6%), type IIIB was 186 of 579 (32.1%), type IIIC was 22 of 579 (3.8%), and type IV was 14 of 579 (2.4%). This huge amount of literature confirms that the anatomy of tricuspid leaflets is extremely variable and that the presence of accessory leaflets is a very common finding (Figure 5).

**Figure 6:**
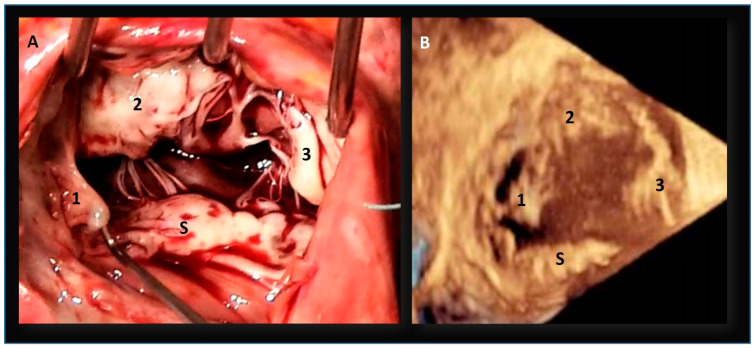
(**A**) Intraoperative image showing the septal leaflet (S) and other three leaflets (1,2,3). (**B**) 3D TEE transgastric view, also showing four leaflets.

The coincidence that accessory leaflets are often localized on the mural annulus (same anatomical architecture is present in the posterior leaflet of the mitral valve) deserves further comment [54,55]. The mural annulus is characterized by significant changes in size and shape during the cardiac cycle. A multi-scalloped leaflet inserted in a high mobile annulus is more suited to better adapt to the annular sphincteric motion, ensuring a more effective closure. Moreover, in a multi-scallop architecture, the short distance between commissures facilitates a complete opening of the scallops during the diastole. Conversely, a nonscalloped C-shaped leaflet and the consequent long intercommissural distance would impede a full opening (Figure 6).

The septal leaflet warrants a more in-depth description. Characteristically, much of the septal leaflet is indeed septal and shows thin chordae arising from the interventricular septum and attaching on the ventricular surface of the leaflet [29]. However, it has an anterior portion that continues away from the septum to be supported by the medial papillary muscle (of Lancisi) at the antero-septal commissure. This portion sometimes appears as a distinct scallop appearing as a separate leaflet.

**Figure 7:**
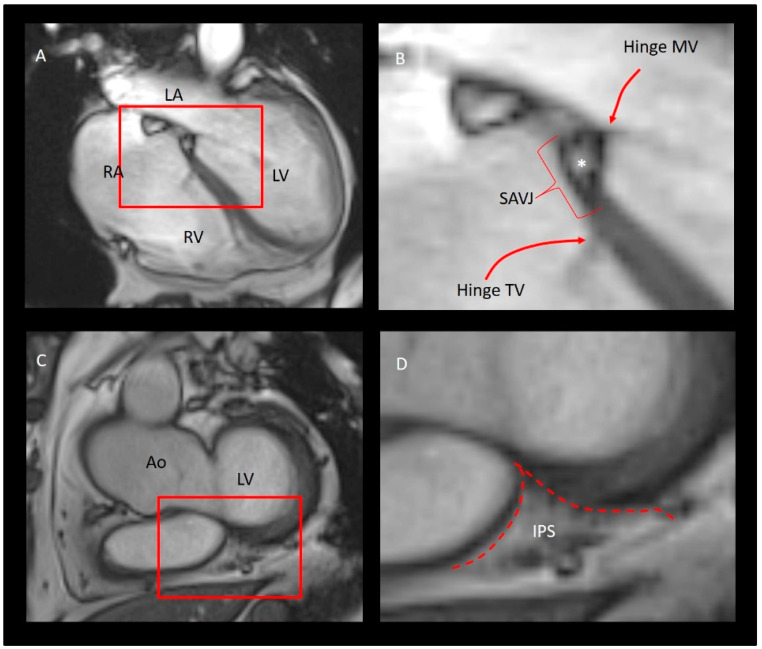
(**A**). CMR four chamber view. (**B**) magnified image of the structure inside the read box. The image shows the septal atrioventricular junction (SAVJ), the hinge line of tricuspid (TV), and mitral (MV) valves. The asterisk points at the leaf of adipose tissue in between the right atrial wall on the right side, the crest of the interventricular septum on left side (see text). (**C**) MRI short-axis view on the base of left ventricle (LV). (**D**) Magnified image showing the inferior pyramidal space (IPS). The red dotted line marks the boundaries of the IPS. RA = right atrium; LA = left atrium; RV = right ventricle; LV = left ventricle; Ao = aorta.

Relative to the short mitral hinge line at the septum, the hinge line of the TV at the septum is longer and is inserted more apically, thus leaving a portion of septum that divides the right atrium from the left ventricle and is named the atrio-ventricular septum (AVS). This septum is a characteristic area where atria and ventricular septa join with the anterior mitral and septal tricuspid leaflets. This region, centrally located in the heart, is named “crux cordis”. This anatomical arrangement was first described by Silverman and Schiller in their seminal paper “Apex Echocardiography” [60]. The AVS is not completely muscular, as once believed; rather, it consists of three structures disposed like a sandwich: the right atrial wall on the right side, the crest of the interventricular septum on left side and a small leaf of adipose tissue (…. representing the “meat” of the sandwich…) in between [61,62]. This adipose space is where the AV nodal artery runs to supply the AV node. While all the three imaging techniques are able to illustrate the atrio-ventricular septum, distinguishing the three-layered assembly depends on the amount of adipose tissue that resides inside. When enough, CMR provides very informative images (Figure 6A,B)). This leaf of adipose tissue is an extension of the “so-called” inferior pyramidal space [63]. This space is in continuity with the posterior-inferior interatrial groove and has a tridimensional aspect of a pyramid (or cone) with the base located in the posterior/inferior surface of the heart and the apex wedging toward the central fibrous body (Figure 7).

Anterior-superiorly, the septal leaflet inserts on the membranous septum (MS). By virtue of the insertion of the septal leaflet, part of this septum is located in atrioventricular position (i.e., separating the right atrium from the left ventricle) while the remainder interposes between the ventricular cavities. When viewed from the left ventricle, the MS almost always lies at the base of the interleaflet fibrous triangle between the right and noncoronary sinuses. Inferiorly, the MS is in continuity with the crest of interventricular septum. In some hearts, the hinge line of septal leaflet is positioned so as to place one-third of the septum in atrioventricular position; in others, the MS is divided half and half between the ventricular and atrioventricular portions [64]. Importantly, the posterior-inferior part of the atrioventricular MS abuts the central fibrous body containing the His bundle that adjoins the atrioventricular node situated on the atrial side, while the continuation of the His bundle, the atrioventricular bundle, runs between the inferior margins of the ventricular MS and the muscular crest of the ventricular septum.

## 7. What Are the Similarities and Differences between the Tricuspid and Mitral Valve

Although exposed to much lower systolic pressure, the general anatomic architecture of the TV is surprisingly similar to that of mitral valve. Both valves are constituted by an annulus, leaflets, chordae and papillary muscles. Both annuli can be divided in two components: one straight component and one C-shaped component. In the mitral valve, the C-shaped component is named posterior annulus and sustains the posterior leaflet, while the C-shaped component of tricuspid annulus is named mural annulus and sustains the anterior-superior and inferior leaflets and/or additional scallops. Both posterior and mural annuli are made up by the convergence of four structures: the atrial and ventricular myocardium, the hinge line of leaflets and the epicardial adipose tissue [29,30]. The only difference between the two C-shaped annuli is that the posterior annulus has a cord of fibrous tissue that “glues” these four components (although incomplete), while the mural annulus does not have any fibrous string. Consequently, the mural annulus is “softer” than the mitral posterior annulus. Sphincteric action and dilation of both annuli are asymmetric exclusively depending on the C-shaped components. Interestingly, while the posterior mitral annulus may be subject to extensive calcifications, in the tricuspid annulus, calcifications are almost absent. Differences are more noticeable between the anterior mitral and septal annuli. Notably, the hinge line of anterior leaflet continues with the mitral-aortic continuity without any macroscopic or microscopic boundary. Moreover, the anterior mitral annulus is reinforced at each extremity by two dense nodules of fibrous tissue called trigons. Conversely, the septal tricuspid annulus is formed by the hinge line of the septal leaflet, and only its anterior part is reinforced by the membranous septum. Both annuli have a saddle-shaped configuration (although in the tricuspid annulus, the saddle is less accentuated and defined). Finally, both annuli tend to become planar once dilated.

Although called bicuspid and tricuspid, the left and right architecture of atrio-ventricular leaflets is almost similar. Indeed, the septal tricuspid leaflet may correspond to the anterior mitral leaflet, while the scalloped posterior leaflet may correspond to leaflets inserted on the mural annulus. Both mitral and tricuspid leaflets have a rough zone which corresponds on the atrial side to the coaptation zone, and in both valves the commissures do not reach the hinge line, leaving a strip of valvular tissue between leaflets and similarly between scallops. Finally, both leaflets have the same three-layered microscopic structure consisting of an atrial layer containing lamellar collagen and elastin sheet, the spongiosa containing loose connective fibers and glycosaminoglycans, and the fibrosa made by robust sheets of collagen fibers. The difference consists only on the fact that in the tricuspid leaflets, layers are thinner. Table 1 shows differences and similarities.

## 8. Conclusions

In this review, we discussed some controversial or still unclear aspects of tricuspid anatomy. In particular, we focused on the anatomical architecture of the so-called tricuspid annulus and on the extreme variability in number of leaflets. This latter point deserves further comment. The term “tricuspid”, referring to the right atrioventricular valve, supposes this valve should have three well-defined leaflets. This assumption does not correspond to the anatomical reality because in a significant percentage of cases, the valve has more than three leaflets. In overcoming this discord, a previous anatomic study of 100 autopsied hearts had indeed proposed that the TV is bicuspid in having a septal leaflet without clefts and a mural leaflet with multiple clefts forming several scallops. [64] We acknowledge, on the other hand, that a detailed description purely based on the anatomical aspects of the individual valve morphology would be extremely complex and not practical. Indeed, only three-dimensional transesophageal echocardiography may allow a precise identification of this huge heterogeneity. Though we are perfectly aware that the term “tricuspid” valve will continue to be maintained in the common language, we believe that labelling the right atrioventricular valve as having a septal and a “mural” leaflet would be simpler and less confounding.

## Figures and Tables

**Table 1:** Similarities and differences between tricuspid and mitral valves.

Tricuspid Valve		Mitral Valve	
***Annulus***	Two components: septal annulus and C-shaped mural annulus.	***Annulus***	Two components: anterior annulus and C-shaped posterior annulus
Mural annulus	C-shaped configuration. Made up of convergence of four tissues: atrial myocardium, ventricular myocardium, hinge line of leaflets and adipose tissue. No fibrous ring. Asymmetric sphincteric contraction of the annulus depending almost exclusively on the mural annulus. The dilation of the annulus mainly affects the mural annulus. No calcifications	Posterior annulus	C-shaped configuration. Made up of convergence of four tissues: atrial myocardium, ventricular myocardium, hinge line of leaflets and adipose tissue. An incomplete fibrous semi-ring glues these components. Asymmetric sphincteric contraction of the annulus depends almost exclusively on the posterior annulus. The dilation of the annulus mainly affects the posterior annulus. Prone to calcification
Septal annulus	Made up of the insertion of septal leaflet with collagen fibers. The hinge line of septal leaflet divides the membranous septum into two parts (see text)	Anterior annulus	Hinge line of anterior leaflet is in continuity with the mitral-aortic curtain. Reinforced by two fibrous nodules (trigons) at the extremities of the mitral-aortic curtain
Saddle shape configuration	Less accentuated both in systole and diastole. The saddle-shaped configuration disappears (the annulus become flat) in annular dilation	Saddle shape configuration	More accentuated both in systole and in diastole. The saddle-shaped configuration disappears (the annulus become flat) in annular dilation
***Leaflets***		***Leaflets***	
	Three main leaflets and three main commissures. Thin and fragile. The commissures do not reach the hinge line, leaving a strip of tissue between leaflets. Two or more leaflets inserted on mural annulus. Each leaflet presents with a basal, a clear and a rough zone. On the atrial side the rough zone corresponds to the coaptation zone. Additional leaflets especially on mural annulus. Three layers (atrialis, spongiosa and fibrosa) thinner that the corresponding mitral leaflets		Two main leaflets and two main commissures. Thick and robust. The commissures do not reach the hinge line, leaving a strip of tissue between leaflets. Three or more scallops on posterior leaflet Each leaflet presents with a clear and a rough zone. On the atrial side the rough zone corresponds to the coaptation zone. Three layers (atrialis, spongiosa and fibrosa) thicker than the corresponding tricuspid leaflets
***Papillary muscles***	Three papillary muscles. Anterior, inferior and septal. The anterior is the dominant arising from the moderator band. Usually the inferior papillary muscles have multiple and thin heads originating from the inferior wall. The septally located papillary muscle, may have multiple heads originating directly from the septum.	***Papillary muscles***	Two groups of papillary muscles: antero-medial and postero-lateral.
